# Suicidal Drownings with Psychiatric Disorders in Shanghai: A Retrospective Study from 2010.1 to 2014.6

**DOI:** 10.1371/journal.pone.0121050

**Published:** 2015-04-27

**Authors:** You-Xin Fang, Meng He, Jun-Yi Lin, Kai-Jun Ma, Hai Zhao, Zhen Hong, Bei-Xu Li

**Affiliations:** 1 Department of Neurology, Huashan Hospital, Fudan University, Shanghai, P. R. China; 2 Department of Forensic Medicine, School of Basic Medical Sciences, Fudan University, Shanghai, P. R. China; 3 Shanghai Key Laboratory of Crime Scene Evidence, Institute of Forensic Science, Shanghai Public Security Bureau, Shanghai, P. R. China; 4 Shanghai Public Security Bureau Marine Police Headquarter, Shanghai Public Security Bureau, Shanghai, P. R. China; University of Vienna, AUSTRIA

## Abstract

Psychiatric disorders exhibited in 13% suicidal drownings in Southwestern Croatia and 63% in Milan, but in China is unknown. This study is committed to outline the feature of a suicidal drowning with psychiatric disorder, show mental status and reveal key factor to high incidence in China. Immersed corpses were handled by SPSBMPH in its jurisdiction range. Half of immersed corpses were suicidal, and nearly half of suicides had psychiatric disorders. 104 suicidal drownings with psychiatric disorders cases from 2010.1 to 2014.6 were reviewed (21.5% of all immersed corpses, 42.1% of suicides). Most victims clothed normally, and only 2 fastened attached weights. Male victims were more and younger than female. Psycho were prone to commit suicidal drowning in warm and hot season. Psycho were prone to choose familiar area to commit suicide, 45 decedents were found in their familiar areas. Suicidal drowings were occult without suicide attempts, suicide note or abnormal clothing, but showed abnormal mental or behavior changes prior to suicide. The three leading psychiatric disorders were depression (33.7%), depression status (30.8%) and schizophrenia (20.2%). Only 44.2% decedents had visited psychiatric disorder specialist, and merely less than 10% patients could adhere to regular medication. No regular medication on psychiatric disorder was the key factor contributing to high incidence of suicide in psycho. Professional psychiatric and psychological intervention should be taken as soon as possible when they had psychiatric symptoms or suffered misfortune. Guardians should be alert to patients’ abnormality to detect their suicidal ideation and intervene, especially in warm season.

## Introduction

Shanghai, located in the Yangtze River Delta, has many rivers, canals, streams and lakes and is known for its rich fresh water resources as part of the Taihu drainage area. Shanghai is also a coastal city. Shanghai has a population over 24 million, which are composed by 14 million locals and 10 million migrants including 0.17 million international migrants. Shanghai Public Security Bureau Marine Police Headquarter (SPSBMPH) is a branch of Shanghai Public Security Bureau (SPSB) which takes responsibility for public security, crime control, emergencies and traffic administration in Shanghai. SPSBMPH’s jurisdiction range is Yangtze River, Suzhou River and their main tributaries in downtown. While seas, water wells, lakes or other rivers were not included. SPSBMPH’s working area is fresh water only. Medical examiners (MEs) in SPSBMPH has same duty and procedure as SPSB to support investigation on unnatural death in its jurisdiction range [1]. SPSBMPH has its own characteristic, because most bodies handled by SPSBMPH are drowned in fresh rivers and died from drowning. Half of these bodies were suicides.

Suicide is a serious international public health problem. In the world, approximately 1 million people killed themselves per year [2]. In China, suicide is the fifth most important cause of death nationwide, and the mean annual suicide rate has been estimated to be 23 per 100,000 individuals, which means about 0.31 million Chinese killed themselves per year [3]. Psychological autopsy studies revealed that about 90% of suicides had a ‘psychiatric disorder’, which contributed to 47–74% of the population risk of suicide [4,5]. Half of the suicides met criteria for depression [4,5]. Besides depression, suicide were reported to be the largest single cause of excess mortality in schizophrenia with suicide rate in schizophrenia 2–10% [6,7,8,9,10,11]. It means that psychiatric condition may be the strongest risk known factor for suicide [4,5].

In Shanghai, more than 100 suicides per year were autopsied by SPSB. Drowning is the fifth cause of suicide, accounting for 7.5% suicides [1]. Similarly, in Milan, Italy, drowning represents the fifth cause of suicide, accounting for 3.8% suicides [12]. And the percentage of suicidal drowning in Southwestern Croatia is higher, accounting for 10% suicides [13]. Psychiatric disorders exhibited in 13% suicidal drownings in Southwestern Croatia and 63.3% in Milan [12,13]. However, the mental status of suicidal drownings in China is unknown. And what contributes to high incidence of suicide in psycho in China is also unknown.

The present study is committed to outline the feature of a suicidal drowning with psychiatric disorder, show mental status and reveal key factor to high incidence in China in order to support investigation on immersion deaths, prevent psycho from suicide, protect their health and call attention on public mental health.

## Materials and Methods

This study was a retrospective review of cases of individuals with psychiatric disorder who committed suicide by drowning in SPSBMPH over a 4.5-year period from 2010.1 to 2014.6.

Cases were accepted for this study according to the following criteria: (1) corpses were found in Yangtze River, Suzhou River and their main tributaries in Shanghai downtown, and handled by SPSBMPH; (2) corpses were identified by DNA techniques; (3) manner of death was suicide; (4) cause of death was drowning; (5) definite or suspected medical history of psychiatric disorder mentioned in investigation on victims; (6) all the records were assessed by two psychiatrists, and psychiatric disorder was confirmed according to The Diagnostic and Statistical Manual of Mental Disorders-IV.

A total of 104 cases met the study criteria of suicidal drowning with psychiatric disorders. Chinese are not used to visiting a psychiatric disorder specialist, and their medical records are not as integral and detailed as those in US or EU, so some information was missed, unrecorded or unknown. We took as much information as we can from these cases’ autopsy archives, initial investigation, police reports, hospital reports and interviews with victims’ family in SPSBMPH. These materials were reviewed and analyzed as to (1) demographic data of the victim, such as age, gender, and registered permanent residence; (2) personal information obtained from interviews with victim’s next of kin, near relatives, community workers and from a reviewing of the medical records, including suicide attempts, abnormal mental or behavior changes prior to suicide, suicide note or information, medical history, regular medication, compliance with medical order, and classification of psychiatric disorders; (3) relationship between body-found location and residence; (4) corpse inspection including degree of decomposition, clothing, attached weights to the bodies, postmortem injuries, and toxicological test and diatoms extraction; (5) quantity of suicide per month, quantity of drowned body found per month, and time of immersion (when these variables were measured, only data from 2010.1 to 2013.12 were used (87 cases) to ensure the season and temperature balanced).

The data were presented as means-standard errors (SE) for age. Statistical analysis was given using the related programs in Microsoft Excel 2013.

This retrospective study was based on the forensic autopsy cases archives. It was approved by both Shanghai Public Security Bureau Ethics Committee and Fudan University Ethics Committee. The written informed consent was given by decedent’s next of kin, who was told that the investigation information might be used in the scientific research when the autopsy was taken. All the archives were anonymized and de-identified prior to analysis.

## Results

SPSBMPH handled 484 immersed bodies from 2010.1 to 2014.6. 247 in 484 bodies were suicidal drownings (51.0%). 104 in 247 suicidal drownings had psychiatric disorders (42.1%). 21.5% of all 484 immersed bodies were suicidal drownings with psychiatric disorders. Besides the 104 suicidal drownings, there was 1 decedent with psychiatric disorder in 484 decedents, whose manner and cause of death were undetermined. A total of 105 immersed decedents had psychiatric disorders in this period, whose manner of death was nearly all suicide (99.0%). 104 victims were all Chinese (one got American nationality).

### General Demographic Characteristics

#### Age and gender (Table 1)

The average age of all the victims was 49.5±19.0 year-old. Male average age 48.1±19.6 year-old was younger than female average age 51.3±18.2 year-old. Male suicides (57) were more than female (47). Male suicides mainly distributed from 20 to 69 year-old, and female suicides mainly distributed from 30 to 79 year-old, 10 years later than male. Male suicides in 20–29 year-old were significantly higher than female in that age group and male in other age groups.

**Table 1:** Age and gender.

Age (year)	Male	Female	Total
10–19	1	2	3
20–29	13	3	16
30–39	9	9	18
40–49	7	7	14
50–59	9	10	19
60–69	9	7	16
70–79	5	7	12
80–89	4	2	6
Total	57	47	104

#### Registered permanent residence

67 locals and 37 migrants composed 104 decedents.

#### Occupations (Table 2)

27 decedents were retired, 18 were unemployed and 16 were office workers. 32 decedents’ occupations were unrecorded or unknown.

**Table 2:** Occupations.

Occupations	Number
Retired	27
Unemployed	18
Office worker	16
Private business owner	4
Student	3
Construction worker	2
Others	2
Unrecorded or unknown	32
Total	104

#### Education (Table 3)

2 decedents’ education status was illiteracy. 9 decedents’ education status was primary school, and 30 decedents’ education status was middle school. Two third decedents (41/62) with definite education status were below high school.

**Table 3:** Education status.

Education status	Number
Illiteracy	2
Primary school	9
Middle school	30
High school	10
University or college	9
Master	1
Doctor	1
Unrecorded or unknown	42
Total	104

#### Marriage

32 decedents were single, 49 were married, 6 were divorced, 10 were widowed and 7 were unrecorded or unknown.

### Corpse Inspection

#### Degree of decomposition (Table 4)

According to the practice, SPSBMPH divided degree of decomposition into 7 levels. Degree of decomposition of 104 suicidal drownings was shown in Table 4. 44 bodies (42.3%) were fresh and 43 bodies (41.3%) were mild decomposed when they were found.

**Table 4:** Degree of decomposition.

Degree of decomposition	Number
Fresh	44
abdominal distention, greenish discoloration on cadaver	8
Decomposed venous rete, putrefactive blister	35
Exfoliation, putrefactive fluid from mouth and nose	10
Bloated cadaver	1
Adipocere formation, soft tissue colliquation	1
Skeletonized	5
Total	104

#### Clothing and attached weights

94 decedents wore normally, while 10 wore abnormally. 9 in 10 decedents only wore tops, or pants, or underwear, even nothing. Besides, 1 decedent wore graveclothes. 2 decedents fastened accessory loads to their neck or ankles, which were fewer than previous literatures [12,13].

#### Postmortem injuries

Injuries by fish occurred in 1 body. Injuries by fish didn’t appear in 102 bodies, and 1 body was undetermined. Injuries by screw propeller occurred in 4 bodies, and didn’t appear in 100 bodies. 99 bodies didn’t show either injuries by fish or injuries by screw propeller.

#### Number of suicide per month, number of immersed body found per month and time of immersion (Fig 1, Table 5)

**Fig 1:**
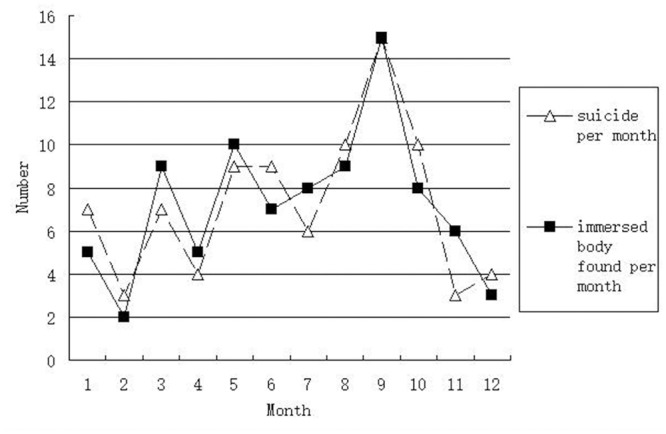
Number of suicide per month and immersed body found per month.

The changes of suicide per month and immersed body found per month were nearly coincident (Fig 1). Numbers of suicide per month and immersed body found per month in warm and hot months were bigger than ones in cold months. In December, January and February, number of suicide per month was bigger than number of immersed body found per month. In March, April and May, number of immersed body found was bigger than number of suicide per month per month.

**Table 5:** Time of immersion.

Time of immersion (day)	Number
0	20
1–2	36
3–7	14
8–30	14
>30	3
Total	87

According to the practice, SPSBMPH divided time of immersion into 5 phrases (Table 5). Time of immersion of 87 bodies was shown in Table 5. 20 bodies (23.0%) were found the same day, and 36 (41.4%) were found within 2 days.

#### Relationship between body-found place and residence

44 decedents were found in the same area as their residences, while 54 were not in the same area as their residences. 6 decedents were unrecorded or unknown. 1 in 54 decedents not in the same area was found near her work place.

#### Toxicological test and diatoms extraction examination

Toxicological test was not taken in 99 bodies. 4 bodies had negative toxicological result, and 1 body used amphetamin chloride before suicide. Diatoms extraction examination was not taken in 100 bodies, and 4 had positive diatom examination results.

### Case Investigation

#### Suicide attempts

18 decedents had had suicide attempts (6 had had multiple attempts), 80 had had no suicide attempts, and 6 were unrecorded or unknown.

#### Abnormal mental or behavior changes prior to suicide (Table 6)

81 decedents had abnormal mental or behavior changes prior to suicide, while 23 were unrecorded or unknown. Weariness of life was most common (29/81, 35.8%). Stress in daily life such as employment pressure, huge debt and family misfortune was the second (9/81, 11.1%). Both love emotional conflict and introversion were the third (6/81, 7.4%).

**Table 6:** Abnormal mental or behavior changes prior to suicide.

Abnormal mental or behavior changes prior to suicide	Number
Weariness of life	29
Stress	9
Love emotional conflict	6
Introversion, silence	6
Depression caused by disease	5
Run away from home	4
Hallucination	4
Delusion of persecution	3
Patient refusal of medication	3
Irritability	2
Unrecorded or unknown	23
Total	104

#### Suicide note or information

24 decedents left suicide note or information prior to death, while 80 left nothing.

### Medical History

#### History of visit psychiatric disorder specialist, treatment and compliance (Table 7)

46 decedents (44.2%) had visited psychiatric disorder specialist, while 44 had not. 20 in 46 decedents took treatment, only 9 decedents (8.7%) stuck to regular treatment. 1 decedent took antidepressants as self-medication.

**Table 7:** History of visit psychiatric disorder specialist, treatment and compliance.

History of visit psychiatric disorder specialist	History of treatment	Compliance
Yes 46	Yes 20	Good 9
		Bad 4
		Unrecorded or unknown 7
	Refuse 2	Bad 2
	No 2	No 2
	Unrecorded or unknown 22	Unrecorded or unknown 22
No 44	No 43	No 43
	Yes 1	No 1
Unrecorded or unknown 14	Unrecorded or unknown 14	Unrecorded or unknown 14

#### Classification of psychiatric disorders (Table 8)

The three leading psychiatric disorders were depression (35, 33.7%), depression status (32, 30.8%) and schizophrenia (21, 20.2%). Senile dementia (9, 8.7%) and anxiety (2, 1.9%) were next.

**Table 8:** Classification of psychiatric disorders.

Classification of psychiatric disorders	Number
Depression	35
Depression status	32
Schizophrenia	21
Senile dementia	9
Anxiety	2
Mental disorders due to brain trauma	1
Mental disorders due to cerebrovascular disease	1
Mental retardation	1
Psychosexual disorder	1
Indefinite	1
Total	104

#### Other diseases

23 decedents had other diseases, 76 didn’t have other diseases and 5 were unrecorded or unknown. In 23 decedents, 4 were stroke, 3 were diabetes mellitus and the rest were sporadic diseases.

#### Information provider

82 decedents’ information was provided by next of kin, 21 by near relative and 1 by community worker.

## Discussion

Male suicides were more and younger than female in this study, with the peak in 20–29 year-old group. Besides male is a recognized general suicide risk factor in psychiatric disorders [14], it may be the reason that love emotional conflicts, employment and economic pressure intensively outbreak in male at this age in China. For instance, 1 depressed doctor killed himself because he could not get a satisfactory job in this study.

The discovery of suicidal drowning committed by young individuals has to alert forensic pathologists and requires careful investigation. Three young victims under 19 year-old were one 13 year-old female, one 16 year-old female and one 18 year-old male. The 16 year-old girl had depression status without seeking medical help. She was introverted and refused communication prior to death. She had left suicide note, which was the most convincing evidence. The 13 year-old girl was a schizophrenic. She met a psychiatrist before, but the treatment and compliance were unknown in the records. She had run away from home five times within one single month prior to death, showing tendency of suicide. The drowning place and discovery place was the same one, which the 13 year-old victim neither familiar with nor went before. The 18 year-old male had major depression with regular medication for two years, but his compliance was unknown. His body was partially skeletonized. He was seen jump into water voluntarily, based on coincidence of his identification and previous record of calling police from a witness.

Two skeletonized corpses were proved by witness of committing suicidal drowning voluntarily as the 18 year-old male. Diatoms extraction test was done in one and got positive result. Investigation showed the other 2 skeletonized corpses had left suicide notes at home after they were identified. And cause of death in two highly decomposed corpses were basically determined by exclusion of foul play, case investigation, and diatoms extraction test whose results were both positive in multiple organs and tissues. And one of them had left suicide note at home. The determination of suicidal drowning in highly decomposed cases required rigorous and reasonable evidences, and should be considered integrally with case investigation. Drowning corpses which cannot met these rigorous and reasonable standards still remain undetermined. Maybe that is why only 7 cases met the criteria in this study.

The information sources of 104 suicidal drownings were reliable. Suicide is strongly correlated with psychiatric disorders, half of the suicides were associated with psychiatric disorders, and almost all the immersed decedents with psychiatric disorders were suicides in this study [15].

Psycho were prone to commit suicidal drowning in warm and hot season. The suicide peak is in September, similar as seasonal pattern in other countries [16,17,18,19,20]. The trend picture of mensile suicides in Shanghai is the same as previous report in Milan, Italy [12].

Psycho were prone to choose familiar area to commit suicide. Nearly half of the discovery places (44/98) were the same area as victims’ residences. Besides, 1 corpse was found near her work place. Though based on the present information, we were not able to detail whether the discovery place or the victim got into water was the area which the victim was familiar with in those corpses found away from residences (53/98); additionally, the fresh river current which is SPSBMPH’s jurisdiction range will influence body location. River current flows gently in Shanghai downtown, and the drowned corpses are usually found shortly. Shift of corpses is not obvious. In our opinion, drowned corpses which were brought from residence place of committing suicide to unfamiliar discovery place were much more than those which were brought from unfamiliar place of committing suicide to residence place. However, consistent with the conclusion that elderly people in Southwestern Croatia were prone to choose act in vicinity of their houses [13], 45 cases made it reasonable to believe the proneness.

104 suicides were occult without suicide attempts, suicide note or abnormal clothing, but showed abnormal mental or behavior changes prior to suicide which were unfortunately neglected by their guardians. Interventions failed to get in promptly.

The three leading psychiatric disorders in suicidal drownings were depression (33.7%), depression status (30.8%) and schizophrenia (20.2%). Previous data showed that patients with affective psychosis had a significantly higher rate of suicidal attempts (15.3%) than those with schizophrenia (7.5%) in China [21]. It was reported that half of the suicides met criteria for depression, and in this study more than half of victims were suffering from depressive disorder [4,5]. Approximately 40% of suicides in China were attributable to depression [3,22]. The suicide rate of depression was reported 17.1% [23]. Besides depression, schizophrenia patients also have a high suicidal tendency. Suicide rate in schizophrenia was 2–10%, and suicide was the largest single cause of death in schizophrenia [6,7,8,9,10,11]. The cumulative risk of suicide attempt was reported 26.3% for major depression and 13.1% for schizophrenia [24]. Depression and schizophrenia are the major factors in suicidal drownings with psychiatric disorders, as well as in suicides with psychiatric disorders.

This study paid additional attention on victims’ treatment and compliance, pointing out that no regular medication on psychiatric disorder was the key factor contributing to high incidence of suicide in psycho in China. Merely less than 10% patients (8.7%, 9/104) could adhere to regular medication after appearance of mental disorders based on the present study. Less than half of the decedents (44.2%, 46/104) had visited psychiatric disorder specialist. Among them, only 20 patients had taken regular medication. However, only 9 out of 20 patients could adhere to treatment in accordance with medical order. Many Chinese are not used to seeking medical help, especially in psychiatric and psychological disorders. They think seeking medical help are unlucky, so doctors and medicine are big taboo of them. In this study, victims were usually not compliant with medical order. The reason why these people lacking medications is their non-compliance.

There are some limitations of this study. SPSBMPH does not have routine procedures of toxicological test and diatoms extraction test as western countries, which is common defect in China’s postmortem examination, so toxicological test and diatoms extraction test were not taken in most cases in the practice [1]. The postmortem blood alcohol level is helpful to determine manner of death, suicide or accident? Although the suicide victims are almost always sober [13], this study cannot prove it due to lack of toxicological test results. Because the location where the victims committed suicidal drowning cannot be confirmed in some cases, proneness of committing location can only be implied indirectly.

The jurisdiction of postmortem examination in Shanghai is decentralized, so the investigation of different cases is distributed in different organizations. We do not have a medical examiner’s office which take responsibility for determining the cause and manner of death and assisting the authorities in the investigation of all unattended, unexpected, or unnatural deaths that occur within certain area. All the suicides with psychiatric disorder including hanging, drug intoxication, jumping from height, and so on were not investigated. So a comprehensively retrospective study on suicides with psychiatric disorder was limited. So was the comparison of corpses in salt water and fresh water, even Shanghai is a coastal region.

Most of these 104 suicidal victims had not visited psychiatric disorder specialist or taken long-term and regular psychiatric treatment in accordance with medical order. It is essential to discover and treat psychiatric disorder promptly. Professional psychiatric and psychological intervention should be taken as soon as possible when they had psychiatric symptoms or suffered misfortune. Most of victims in this study did have suicide attempts, or leave suicide information, but most of victims had abnormal mental or behavior changes prior to suicide, suggesting guardians to be alert to their abnormality in order to detect their suicidal ideation and intervene, especially in warm and hot season.

## References

[1] He M, Li WC, Sun DM, Ma KJ, Zhao ZQ, Li BX, et al Epitome of China's Unnatural Deaths: A Historically Retrospective Study of Forensic Autopsy Cases in Shanghai Public Security Bureau From 1990 to 1999. Am J Forensic Med Pathol. 2014; 35: 218–221. 10.1097/PAF.0000000000000115 25084321

[2] Lopez-Morinigo JD, Fernandes AC, Chang CK, Hayes RD, Broadbent M, Stewart R, et al Suicide completion in secondary mental healthcare: a comparison study between schizophrenia spectrum disorders and all other diagnoses. BMC Psychiatry. 2014; 14: 213 10.1186/s12888-014-0213-z 25085220 PMC4149212

[3] Phillips MR, Li X, Zhang Y. Suicide rates in China, 1995–99. Lancet. 2002; 359: 835–840. 11897283 10.1016/S0140-6736(02)07954-0

[4] Cavanagh JT, Carson AJ, Sharpe M, Lawrie SM. Psychological autopsy studies of suicide: a systematic review. Psychol Med. 2003; 33: 395–405. 12701661 10.1017/s0033291702006943

[5] Hawton K, Harriss L, Hall S, Simkin S, Bale E, Bond A. Deliberate self-harm in Oxford, 1990–2000: a time of change in patient characteristics. Psychol Med. 2003; 33: 987–995. 12946083 10.1017/s0033291703007943

[6] Brown S. Excess mortality of schizophrenia. A meta-analysis. Br J Psychiatry. 1997; 171: 502–508. 9519087 10.1192/bjp.171.6.502

[7] Caldwell CB, Gottesman II. Schizophrenics kill themselves too: a review of risk factors for suicide. Schizophr Bull. 1990; 16: 571–589. 2077636 10.1093/schbul/16.4.571

[8] Dutta R, Murray RM, Allardyce J, Jones PB, Boydell JE. Mortality in first-contact psychosis patients in the U.K.: a cohort study. Psychol Med. 2012; 42: 1649–1661. 10.1017/S0033291711002807 22153300

[9] Miles CP. Conditions predisposing to suicide: a review. J Nerv Ment Dis. 1977; 164: 231–246. 321725 10.1097/00005053-197704000-00002

[10] Palmer BA, Pankratz VS, Bostwick JM. The lifetime risk of suicide in schizophrenia: a reexamination. Arch Gen Psychiatry. 2005; 62: 247–253. 15753237 10.1001/archpsyc.62.3.247

[11] Saha S, Chant D, McGrath J. A systematic review of mortality in schizophrenia: is the differential mortality gap worsening over time? Arch Gen Psychiatry. 2007; 64: 1123–1131. 17909124 10.1001/archpsyc.64.10.1123

[12] Muccino E, Crudele GD, Gentile G, Marchesi M, Rancati A, Zoja R. Suicide drowning in the non-coastal territory of Milan. Int J Legal Med. 2014 11 15 10.1007/s00414-014-1115-9 25398634

[13] Stemberga V, Bralic M, Coklo M, Cuculic D, Bosnar A. Suicidal drowning in Southwestern Croatia: a 25-year review. Am J Forensic Med Pathol. 2010; 31: 52–54. 10.1097/PAF.0b013e3181c215c8 19918158

[14] Hawton K, Sutton L, Haw C, Sinclair J, Deeks JJ. Schizophrenia and suicide: systematic review of risk factors. Br J Psychiatry. 2005; 187: 9–20. 15994566 10.1192/bjp.187.1.9

[15] Harris EC, Barraclough B. Suicide as an outcome for mental disorders—A meta-analysis. Br J Psychiatry. 1997; 170: 205–228. 9229027 10.1192/bjp.170.3.205

[16] Ajdacic-Gross V, Wang J, Bopp M, Eich D, Rossler W, Gutzwiller F. Are seasonalities in suicide dependent on suicide methods? A reappraisal. Soc Sci Med. 2003; 57: 1173–1181. 12899902 10.1016/s0277-9536(02)00493-8

[17] Bando DH, Volpe FM. Seasonal variation of suicide in the city of Sao Paulo, Brazil, 1996–2010. Crisis. 2014; 35: 5–9. 10.1027/0227-5910/a000222 23871952

[18] Cantor CH, Hickey PA, De Leo D. Seasonal variation in suicide in a predominantly Caucasian tropical/subtropical region of Australia. Psychopathology. 2000; 33: 303–306. 11060513 10.1159/000029162

[19] Grjibovski AM, Kozhakhmetova G, Kosbayeva A, Menne B. Associations between air temperature and daily suicide counts in Astana, Kazakhstan. Medicina (Kaunas). 2013; 49: 379–385. 24509149

[20] Law CK, De Leo D. Seasonal differences in the day-of-the-week pattern of suicide in Queensland, Australia. Int J Environ Res Public Health. 2013; 10: 2825–2833. 10.3390/ijerph10072825 23880724 PMC3734460

[21] Ran MS, Chan CLW, Xiang MZ, Wu GH. Suicide attempts among patients with psychosis in a Chinese rural community. Acta Psychiatr Scand. 2003; 107: 430–435. 12752019 10.1034/j.1600-0447.2003.02014.x

[22] Cheung YB, Law CK, Chan B, Liu KY, Yip PS. Suicidal ideation and suicidal attempts in a population-based study of Chinese people: risk attributable to hopelessness, depression, and social factors. J Affect Disord. 2006; 90: 193–199. 16406046 10.1016/j.jad.2005.11.018

[23] Jeon HJ, Park JI, Fava M, Mischoulon D, Sohn JH, Seong S, et al Feelings of worthlessness, traumatic experience, and their comorbidity in relation to lifetime suicide attempt in community adults with major depressive disorder. J Affect Disord. 2014; 166: 206–212. 10.1016/j.jad.2014.05.010 25012433

[24] Shibre T, Hanlon C, Medhin G, Alem A, Kebede D, Teferra S, et al Suicide and suicide attempts in people with severe mental disorders in Butajira, Ethiopia: 10 year follow-up of a population-based cohort. BMC Psychiatry. 2014; 14: 150 10.1186/1471-244X-14-150 24886518 PMC4052808

